# Optimizing surgical strategies for retroperitoneal liposarcoma: a comprehensive evaluation of standardized aggressive surgical policies

**DOI:** 10.1186/s12885-024-12629-4

**Published:** 2024-07-18

**Authors:** Dao-Ning Liu, Liang Yan, Zhong-Wu Li, Hai-Yue Wang, Xiu-Yun Tian, Ang Lv, Chun-Yi Hao

**Affiliations:** 1 Key laboratory of Carcinogenesis and Translational Research (Ministry of Education/Beijing), Department of Hepato-Pancreato-Biliary Surgery/Sarcoma center, Peking University Cancer Hospital & Institute, No. 52 Fucheng Road, Haidian District, Beijing, People’s Republic of China; 2 Key laboratory of Carcinogenesis and Translational Research (Ministry of Education/Beijing), Department of Pathology, Peking University Cancer Hospital & Institute, Beijing, People’s Republic of China

**Keywords:** Liposarcoma, Prognostic factors, Surgical procedures, Retroperitoneum, Surgical indications

## Abstract

**Background:**

Retroperitoneal liposarcoma (RLPS) constitutes the majority of retroperitoneal sarcomas. While surgical resection remains the sole curative approach, determining the optimal surgical strategy for RLPS remains elusive. This study addresses the ongoing debate surrounding the optimal surgical strategy for RLPS.

**Methods:**

We recruited 77 patients with RLPS who underwent aggressive surgical policies. Patients were categorized into three surgical subtypes: suprapancreatic RLPS, pancreatic RLPS, and subpancreatic RLPS. Our standardized surgical strategy involved resecting macroscopically uninvolved adjacent organs according to surgical subtypes. We collected clinical, pathological and prognostic data for analyses.

**Results:**

The median follow-up was 45.5 months. Overall survival (OS) and recurrence-free survival (RFS) were significantly correlated with multifocal RLPS, pathological subtype, recurrent RLPS and histological grade (P for OS = 0.011, 0.004, 0.010, and < 0.001, P for RFS = 0.004, 0.001, < 0.001, and < 0.001, respectively). The 5-Year Estimate OS of well-differentiated liposarcoma (WDLPS), G1 RLPS, de novo RLPS and unifocal RLPS were 100%, 89.4%, 75.3% and 69.1%, respectively. The distant metastasis rate was 1.4%. The morbidity rates (≥ grade III) for suprapancreatic, pancreatic, and subpancreatic RLPS were 26.7%, 15.6%, and 13.3%, respectively. The perioperative mortality rate is 2.6%.

**Conclusions:**

Standardized aggressive surgical policies demonstrated prognostic benefits for RLPS, particularly for G1 RLPS, WDLPS, unifocal RLPS, and de novo RLPS. This approach effectively balanced considerations of adequate exposure, surgical safety, and thorough removal of all fat tissue. G1 RLPS, WDLPS, unifocal RLPS, and de novo RLPS could be potential indications for aggressive surgical policies.

## Introduction

Liposarcoma comprises the majority of retroperitoneal sarcomas, including well-differentiated liposarcoma (WDLPS), dedifferentiated liposarcoma (DDLPS), and pleomorphic liposarcoma (PLPS) [1]. Currently, surgical resection is the sole curative approach for retroperitoneal liposarcoma (RLPS) [2]. However, the optimal surgical strategy for RLPS remains unclear [3], and an inappropriate approach may lead to unnecessary resection of unaffected organs, unfavorable prognoses, and increased morbidity and mortality.

The primary goal of RLPS surgery is complete resection, ideally as a single, intact specimen encompassing all involved contiguous organs. This often involves en bloc resection of adjacent organs, especially when obvious invasion is present, frequently involving the colon and kidney. However, proponents of extended resection argue for en bloc resection of seemingly macroscopically uninvolved adjacent organs [3]. Critics of extended resection express concerns about the high rate of multicentric disease and the location of recurrences. Extended resections in a significant percentage of patients may not cover potential multi-local disease recurrence and may only increase the procedure’s morbidity [4]. The extent of resection has been a topic of intense debate.

This study aimed to assess the benefits and drawbacks of adopting aggressive surgical policies for RLPS, with a specific focus on the resection of macroscopically uninvolved adjacent organs. Furthermore, we emphasized the importance of personalizing the surgical strategy for RLPS, considering both the tumor’s characteristics and the patient’s overall condition. Our goal was to explore potential indications for aggressive surgical policies and investigate whether extended resection could offer a survival benefit for RLPS.

## Methods

### Patients

In this study, a total of 77 patients diagnosed with RLPS were included. Ethical approval and written informed consent were obtained from all participants, and patient anonymity has been strictly maintained. Pelvic liposarcoma was excluded due to the difficulty in defining a standardized surgical procedure, and extended resection was deemed unfeasible. The patients included in this study were evaluated and determined to be candidates for R0/1 resection. None of the patients underwent adjuvant/neoadjuvant chemotherapy or radiotherapy, as there is insufficient evidence supporting their efficacy in improving the survival of resectable RLPS [5–8]. Unresectable RLPS that were converted to resectable tumors by preoperative chemotherapy or radiotherapy were not included in this study. The histological grade of RLPS was reassessed using the FNCLCC system by two experienced pathologists who were blinded to the clinical and prognostic information of the patients [9].

### Surgical procedure

**Fig. 1:**
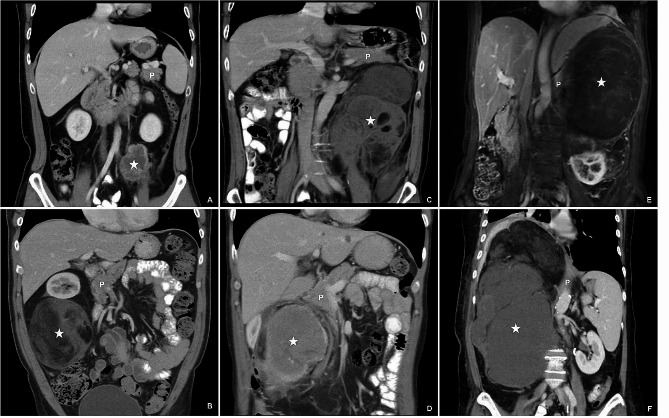
**A** and **B** show subpancreatic RLPS. **C** and **D** show pancreatic RLPS. **E** and **F** show suprapancreatic RLPS. The asterisk denotes the location of RLPS. P pancreas

The study adopted a frontline extended surgical approach and implemented aggressive surgical policies. All patients underwent surgery with the aim of achieving complete resection as a single, intact specimen that included adjacent organs, as delineated by various surgical subtypes outlined in Table 1, and all fat tissue in either the right-sided or left-sided retroperitoneal cavity. The demarcation between the right-sided and left-sided retroperitoneal cavity was defined by the inferior vena cava (IVC). Patients with RLPS were classified into three surgical subtypes based on the relationship between RLPS and the pancreas, as illustrated in Fig. 1. Surgical strategy was determined according to the surgical subtype.

**Table 1:** Surgical subtype of retroperitoneal liposarcoma

	Organs which are highly recommended for en bloc resection with RLPS	
	Left-sided RLPS	Right-sided RLPS
**Subpancreatic type (**Fig. 1A **and B**)	Left kidney; Left adrenal gland; Descending colon	Right kidney; Right adrenal gland; Ascending colon; Distal ileum
**Pancreatic type** (Fig. 1C **and D**)	Left kidney; Left adrenal gland; Descending colon; Pancreatic body and tail; Spleen	Right kidney; Right adrenal gland; Ascending colon; Distal ileum; Pancreaticoduodenectomy*
**Suprapancreatic type** (Fig. 1E **and F**)	Left kidney; Left adrenal gland; Descending colon; Pancreatic body and tail; Spleen; Stomach*	Right kidney; Right adrenal gland; Ascending colon; Distal ileum; Pancreaticoduodenectomy*; Liver*

Organs marked with an asterisk (*) should be meticulously assessed for invasion by RLPS. They will be resected when obvious invasion is present. Other organs will be resected irrespective of the presence or absence of obvious organ invasion

### Stage 1: incision and exploration, Assessment of Surgical subtypes

**Fig. 2:**
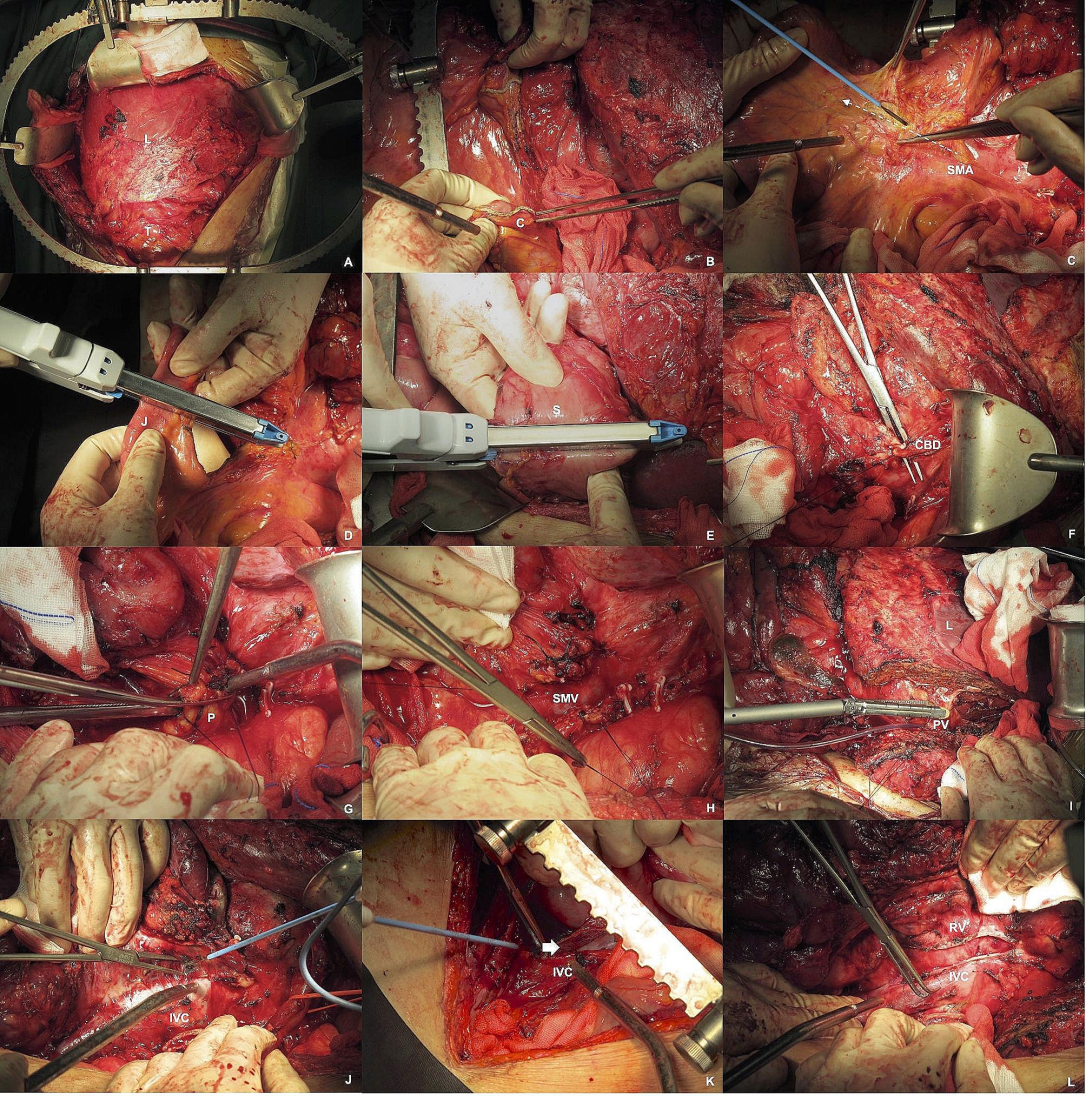
A-L show the surgical procedure of RLPS. L liver; T tumor; C colon; SMA superior mesenteric artery; J jejunum; S stomach; CBD common bile duct; P pancreas; SMV superior mesenteric vein; PV portal vein; HV hepatic vein; IVC inferior vena cava; RV renal vein

An appropriate incision is selected for optimal exposure, including a midline incision, thoracoabdominal incision (for certain suprapancreatic RLPS, Fig. 2A), or abdominal-inguinal incision (for certain subpancreatic RLPS). Following exploration of the entire abdominopelvic cavity, the surgical strategy is determined based on different surgical subtypes, with anteroposterior dissection in the midline.

### Stage 2: Division of the transverse Colon and Mesocolon, dissection of Mesentery

Initially, the transverse colon and mesocolon are divided for both left-sided and right-sided RLPS (Fig. 2B). Whenever possible, the middle colic vessels are spared. Subsequently, the mesentery is dissected along the superior mesenteric artery (SMA) to remove the right part of the small intestine for right-sided RLPS (Fig. 2C). Resection of the small intestine is unnecessary for left-sided RLPS, and resection of the proximal jejunum is only considered when obvious invasion is observed.

### Stage 3 option A: organs resected for right-sided RLPS and IVC exposure

For some right-sided pancreatic and suprapancreatic RLPS, pancreaticoduodenectomy is performed, involving the division of the proximal jejunum, stomach, common bile duct, pancreas neck, and uncinate process of the pancreas (Fig. 2D-H). Right hemi-hepatectomy is conducted for some right-sided suprapancreatic RLPS, involving the liver hanging maneuver. Liver parenchyma, right hepatic pedicle and right hepatic vein would be divided as shown in Fig. 2I. The IVC is fully exposed after the division of the uncinate process of pancreas from SMA and SMV and right hemi-hepatectomy (Fig. 2J and K). The Kocher maneuver and Cattell-Braasch maneuver are executed for IVC exposure of subpancreatic RLPS.

### Stage 3 option B: organs Resected for Left-Sided RLPS and IVC exposure

For left-sided pancreatic and suprapancreatic RLPS, distal pancreatomy and splenectomy are performed, with total gastrectomy for some suprapancreatic RLPS. The distal transection point on the colon, pancreatic neck, and splenic vessels are divided. IVC exposure follows these procedures. The pancreas, spleen, and splenic vessels are liberated and medialized by dissecting close to their borders for IVC exposure of subpancreatic RLPS.

### Stage 4: resection of kidney, adrenal gland and other possible organs

Full exposure of normal IVC above and below the tumor is essential (Fig. 2J, K). Subsequent dissection along the IVC allows for right or left nephrectomy and adrenalectomy (Fig. 2L). Consideration for resection of the ovary, diaphragm, aorta, IVC and other possible organs is given only when evident invasion by RLPS is observed. Routine resection of these organs does not contribute to exposure, surgical safety, and the removal of fat tissue.

### Stage 5: peritonectomy and partial resection of the Psoas muscle

**Fig. 3:**
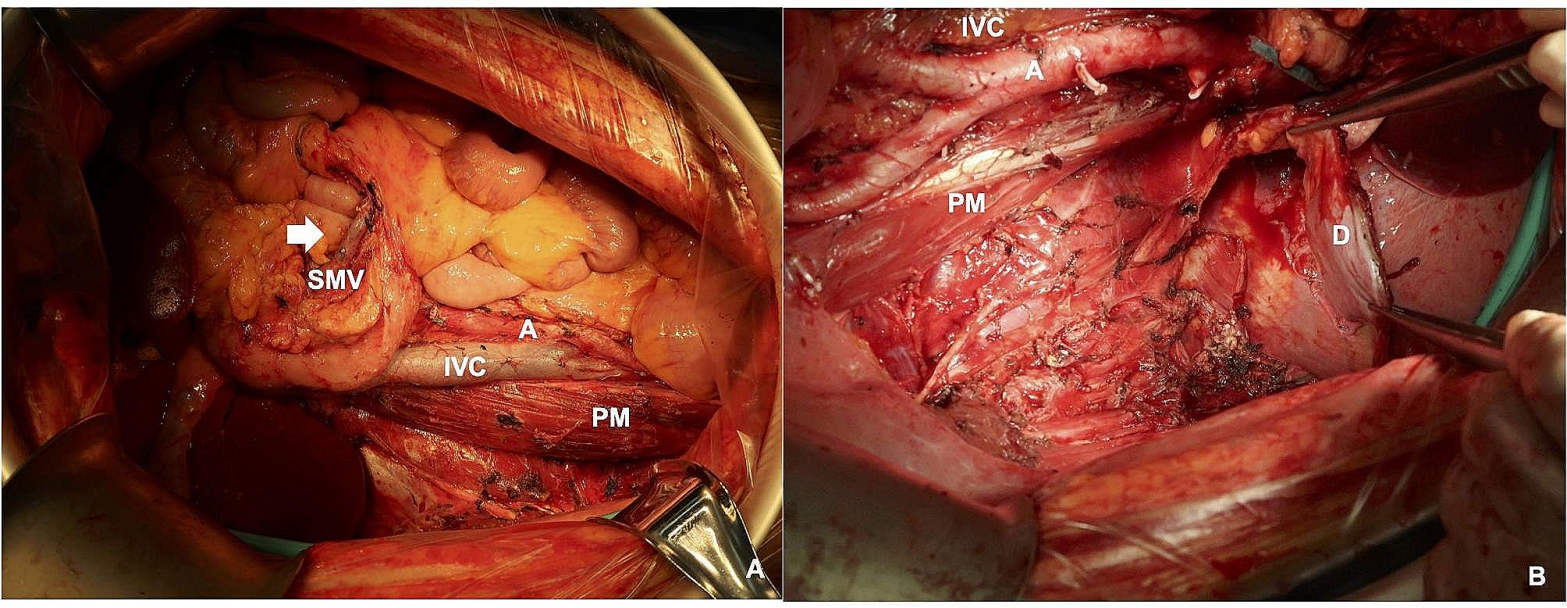
A shows the right-sided surgical field after removal of RLPS. B shows the left-sided surgical field after removal of RLPS. SMV superior mesenteric vein; IVC inferior vena cava; A aorta; PM psoas muscle; D diaphragm

The lateral abdominal wall peritoneum is incised, and dissection continues until reaching the psoas fascia. The fascia is opened, and the femoral nerve is identified and protected. Partial resection of the psoas muscle ensures en bloc resection of all fat tissue in the left-sided or right-sided retroperitoneal cavity. To ensure thorough locoregional control, clearance of all retroperitoneal and intra-abdominal (including mesenteric) fat tissue is carried out. Identification and distal division of the ureter follow in this space. Subsequently, the tumor is removed from the body cavity, leaving no residual fat tissue in the surgical field, as illustrated in Fig. 3.

### Statistics

Data collection and statistical analyses were conducted using IBM SPSS Version 26 (SPSS Inc, Chicago, IL, USA). Enumeration data were presented as mean and standard deviation, while ranked data were analyzed through cross-tabulation and percentages. Recurrence-free survival (RFS) and overall survival (OS) were estimated using the Kaplan-Meier method, with calculations starting from the date of surgery to the date of recurrence and mortality. Statistical differences in OS and RFS were determined by the log-rank test. Various statistical methods were employed, including the t-test, linear regression, Pearson correlation analysis, ANOVA, non-parametric test, chi-square test, and log-rank test. All tests were two-sided, and the threshold for statistical significance was set at *P* ≤ 0.05.

## Results

The entire study population comprised 77 patients who underwent surgery at the Sarcoma Center of Peking University Cancer Hospital over an eight-year period, spanning from November 2013 to July 2022. The overall median follow-up duration was 45.5 months. All patients received R0/1 resection. Eight patients were excluded from the prognostic analysis due to postoperative complications (two patients died of hepatic failure and intestinal fistula), loss of follow-up (three patients) and non-tumor-related causes of death (one patient). Two cases resulted in death from severe malnutrition several months after surgery, possibly linked to multivisceral resection. The clinical characteristics of the patients are outlined in Table 2.

**Table 2:** Clinical characters of RLPS in this cohort

Parameters		*n*	%
**Age(y)**		54.81 ± 10.59	
**Operative time (min)**		512.01 ± 167.83	
**Blood loss (ml)**		2317.14 ± 2580.99	
**Length of stay (days)**		33.26 ± 16.84	
**Tumor size (cm)**		24.45 ± 11.26	
**Resection of organs**		7.34 ± 2.68	
**Organs invaded by sarcoma**		1.42 ± 1.61	
**Gender**			
	Male	43	55.8%
	Female	34	44.2%
**Pathological subtype**			
	WDLPS	22	28.6%
	DDLPS	45	58.4%
	PLPS	10	13.0%
**Histological grade (FNCLCC)**			
	G1	22	28.6%
	G2	39	50.6%
	G3	16	20.8%
**Complication (Dindo–Clavien Classification)**			
	I	18	23.4%
	II	46	59.7%
	III	10	12.9%
	IV	1	1.3%
	V	2	2.6%
**Multifocal RLPS**			
	Yes	15	19.5%
	No	62	80.5%
**Recurrent RLPS**			
	Yes	27	35.1%
	No	50	64.9%
**Vascular resection**			
	Yes	19	24.7%
	No	58	75.3%
**Surgical subtype**			
	Subpancreatic RLPS	30	38.9%
	Pancreatic RLPS	32	41.6%
	Suprapancreatic RLPS	15	19.5%

### Local recurrence and distant metastasis

**Fig. 4:**
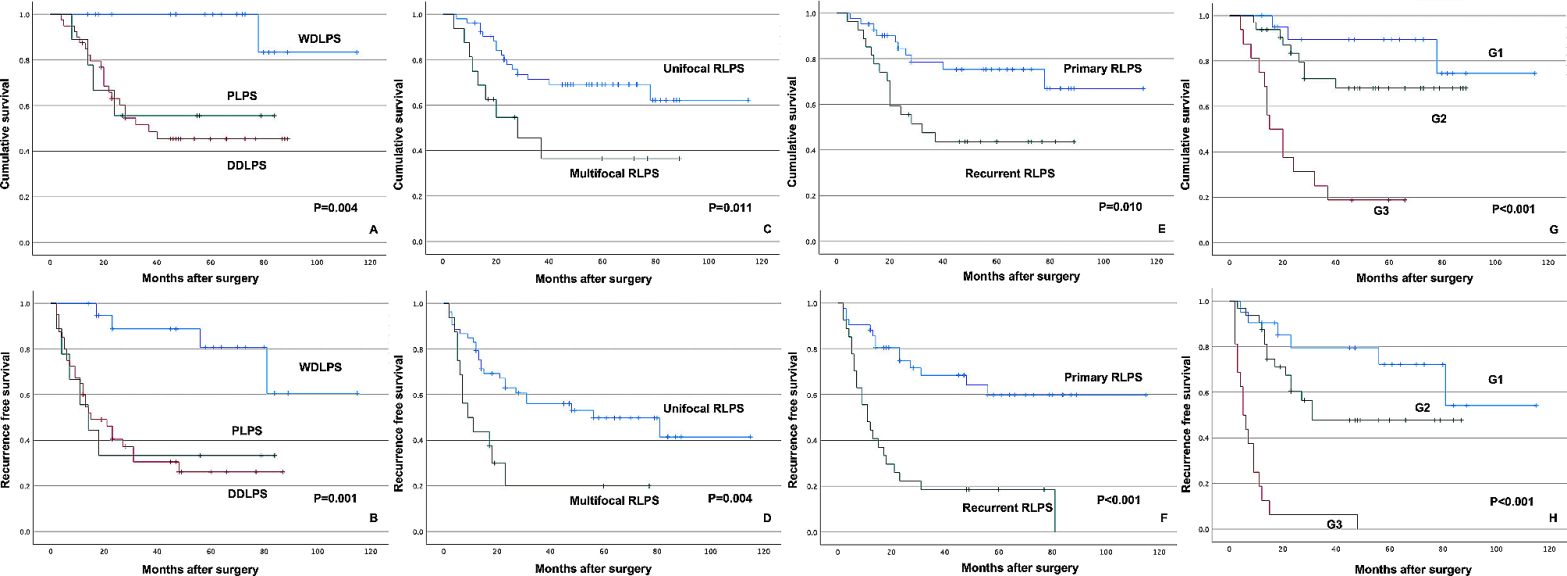
A-H shows the Kaplan–Meier survival curves for multifocal RLPS, pathological subtype, recurrent RLPS, and FNCLCC histological grade

Ovarian metastasis occurred in one patient with DDLPS, resulting in a metastatic incidence of 1.4%. Significant correlations with RFS were observed for multifocal RLPS, pathological subtype, recurrent RLPS, and FNCLCC histological grade using the Kaplan–Meier method (*P* = 0.004, 0.001, < 0.001, and < 0.001, respectively, Fig. 4). Subgroup analysis for 5-year RFS based on grade, histological type, multifocal RLPS, or recurrent RLPS is presented in Table 3. Tumor size (> 20 cm or not) and surgical subtype did not exhibit a correlation with RFS (*P* = 0.246 and 0.118, respectively).

### Overall survival

Multifocal RLPS, pathological subtype, recurrent RLPS, and FNCLCC histological grade showed significant correlations with OS using the Kaplan–Meier method (*P* = 0.011, 0.004, 0.010, and < 0.001, respectively, Fig. 4). Subgroup analysis for 5-year OS based on grade, histological type, multifocal RLPS, or recurrent RLPS is presented in Table 3. Tumor size (> 20 cm or not) and surgical subtype did not correlate with OS (*P* = 0.705 and 0.304, respectively). Multivariable Cox regression analyses were conducted for OS, utilizing potential risk factors identified in univariable analyses. The significant determinants for survival in multivariable analysis were histologic grade and pathological subtype (*P* = 0.001 and 0.022, respectively).

**Table 3:** Overall survival and recurrence free survival of histological grade, histotype, multifocal RLPS and recurrent RLPS

End Point by Subgroup	Overall survival (OS)			Recurrence free survival (RFS)			
	5-Year Estimate (%)	OS (months)	95% CI	5-Year Estimate (%)	RFS (months)		95% CI
Histological grade							
**I**	89.4	99.3	83.4 to 115.2	72.3	83.7		63.6 to 103.9
**II**	68.1	68.0	56.6 to 79.5	47.8	51.0		38.1 to 63.9
**III**	18.8	25.9	15.6 to 36.2	0	8.9		3.5 to 14.4
**Histological subtype**							
**WDLPS**	100	108.8	97.8 to 119.9	80.7	92.8	74.4 to 111.1	
**PLPS**	55.6	53.6	31.2 to 75.9	33.3	34.2	11.0 to 57.4	
**DDLPS**	45.5	51.6	40.3 to 62.9	20	33.7	22.7 to 44.7	
**Unifocal RLPS**	69.1	83.4	70.7 to 96.2	49.8	63.8	49.6 to 78.0	
**Multifocal RLPS**	36.5	43.8	25.1 to 62.6	26.2	23.6	9.0 to 38.2	
**De novo RLPS**	75.3	88.7	74.8 to 102.7	59.9	78.1	62.6 to 93.5	
**Recurrent RLPS**	43.7	49.3	35.8 to 62.9	18.5	23.8	5.5 to 12.9	

### Morbidity and mortality

In the context of right-sided retroperitoneal LPS, a Whipple procedure is performed in 47.5% of cases. 75.7% of left-sided retroperitoneal LPS cases undergo distal pancreatectomy. The morbidity rates (≥ grade III) for right-sided and left-sided RLPS are 16.2% and 9.1%, respectively. Furthermore, the morbidity rates (≥ grade III) of suprapancreatic, pancreatic, and subpancreatic RLPS are 26.7%, 15.6%, and 13.3%, respectively. The morbidity rates of suprapancreatic RLPS is significantly higher than pancreatic and subpancreatic RLPS (*P* = 0.002). Two patients died of surgical complications—one with the suprapancreatic type and another with the pancreatic type, resulting in a perioperative mortality rate of 2.6%. Importantly, no deaths occurred during the surgical procedure.

## Discussion

RLPS has long been recognized for its resistance to conventional chemotherapy and radiotherapy, underscoring the significance of surgical intervention as the primary treatment. However, the surgical approach is notably individualized, considering both tumor characteristics and patient factors [4]. The extent of resection, particularly the debated en bloc resection of adjacent organs that appear macroscopically uninvolved, has been a focal point of discussion [3]. Given the variability in surgical strategies and the subjective assessment of marginal status, accurately predicting the prognosis of RLPS remains challenging. Moreover, the absence of specific criteria for selecting patients for extended resection in previous studies may introduce selection bias [10]. Our study introduces a standardized surgical strategy based on different surgical subtypes, allowing for a comprehensive evaluation of the advantages and disadvantages of aggressive surgical policies for RLPS through long-term follow-up.

Our findings highlight RLPS’s unique clinical challenge, characterized by a minimal metastatic rate but a notable inclination toward local recurrence. In contrast to Alessandro Gronchi’s study reporting an 11% metastatic incidence in RLPS [11], our study observes a 1.4% metastatic incidence. This aligns with the 1.9% reported by Hsuan-Ying Huang and challenges the notion that certain RLPS subtypes may exhibit slightly elevated distant metastasis incidence, such as dedifferentiated liposarcoma and grade 3 RLPS [11–13]. Given these findings, we assert that prioritizing local control is paramount for RLPS, and aggressive surgical policies currently offer the most effective solution.

The optimal RLPS surgical strategy should balance considerations of adequate exposure, surgical safety, and thorough removal of all fat tissue. Extended resection of adjacent organs and fat tissue emerges as the primary approach for achieving local control. Dario Callegaro’s study demonstrated the feasibility of complete clearance of ipsilateral retroperitoneal fat tissue through a frontline extended surgical approach [14]. We contend that insufficient exposure and preservation of certain organs may lead to incomplete resection of retroperitoneal cavity fat tissue, a key factor in local recurrence. To address this, we have devised a grouping method for RLPS, modified the frontline extended surgical approach, and standardized aggressive surgical policies. RLPS, known for its substantial tumor volume, often results in a confined operating space, uncontrollable hemorrhage, and a high postoperative complication rate. Our approach, starting anteroposterior midline dissection, effectively minimizes surgical difficulty, controls blood supply to RLPS and adjacent organs, and maintains numerically comparable morbidity and mortality rates to previous studies [15, 16]. It is important to highlight that two patients died of severe malnutrition several months post-surgery, potentially associated with multivisceral resection. Vigilant emphasis on nutritional support for patients following aggressive surgical interventions is imperative.

While some advocate for en bloc resection of adjacent organs appearing macroscopically uninvolved, critics express concerns about the high incidence of multicentric disease and recurrence locations. Extended resections in a substantial percentage of patients may not encompass potential multi-focal disease recurrence and could only increase the morbidity of the procedure [4]. The 5-year OS and 5-year RFS were reported in the EORTC-62,092 trial (STRASS), off-trial (STREXIT), and Dario Callegaro’s study as ranging from 40 to 71% and 43–76%, respectively. The 5-year OS and 5-year RFS for WDLPS and G1 RLPS were reported as ranging from 87 to 90% and 77–81%, respectively [6, 7, 14]. Our study indicates that aggressive surgical policies result in a numerically higher 5-year OS rate, particularly for G1 RLPS, WDLPS, unifocal RLPS, and de novo RLPS. Conversely, G2/3 RLPS, DDLPS, PLPS, multifocal RLPS, and recurrent RLPS yield equal or lower rates than before. This suggests that G1 RLPS, WDLPS, unifocal RLPS, and de novo RLPS could be potential indications for aggressive surgical policies. Preoperative assessments of pathological subtype, histological grade, recurrent disease, and multifocal disease presence is highly probable to guide precise, personalized surgical decisions. Multivariable Cox analysis uncovers correlations between pathological subtype, FNCLCC histological grade, and OS. Notably, no relationship is identified between tumor size, OS, and RFS. This outcome may be attributed to the aggressive surgical policies implemented in the cohort, ensuring R0/1 resection and precluding an examination of margin status influence.

We performed an average of 7.34 ± 2.68 organ resections, despite the pathological invasion of adjacent organs being only 1.42 ± 1.61 on average. This lack of correlation may be attributed to our aggressive surgical approach. Intraoperatively, distinguishing liposarcoma from normal fat can pose a challenge for surgeons. Notably, the assessment of margin status in RLPS frequently depends on subjective judgments made by the surgeons. Currently, the sole solution to address this issue is through the implementation of aggressive surgical policies. Introducing an objective, real-time margin status assessment is imperative for refining surgical approaches and avoiding unnecessary resections of healthy organs. Our future research will delve into this innovative margin status assessment.

Acknowledging the study limitations is important. While G1 RLPS, WDLPS, unifocal RLPS, and de novo RLPS showcased significant benefits from aggressive surgical policies, the efficacy for other RLPS subtypes remains unknown. The unfavorable prognosis of other RLPS subtypes does not necessarily imply the failure of aggressive surgical policies. For G2/3 RLPS, DDLPS, PLPS, multifocal RLPS, and recurrent RLPS, further studies should investigate whether en bloc resection of adjacent organs with obvious invasion could provide a prognosis comparable to that of this cohort. The ongoing exploration of this hypothesis is imperative for a comprehensive understanding of RLPS surgical strategies.

## Conclusion

Standardized aggressive surgical policies demonstrated prognostic benefits for RLPS, particularly for G1 RLPS, WDLPS, unifocal RLPS, and de novo RLPS. This approach effectively balanced considerations of adequate exposure, surgical safety, and thorough removal of all fat tissue. G1 RLPS, WDLPS, unifocal RLPS, and de novo RLPS could be potential indications for aggressive surgical policies. However, further investigation is necessary to assess the efficacy of these policies for other RLPS subtypes.

## Data Availability

The datasets generated and analysed during the current study are not publicly available due consideration of medical ethics, but are available from the corresponding author on reasonable request.

## Declarations

### Synopsis

We standardized aggressive surgical strategy for retroperitoneal liposarcoma (RLPS) involved resecting macroscopically uninvolved adjacent organs according to surgical subtypes. This approach offered prognostic benefits for RLPS, particularly for well-differentiated, G1, unifocal, and de novo RLPS.
